# Supplementary material for: Physicochemical, Rheological, and Nutritional Quality of Artisanal Fermented Milk Beverages with Cupuassu (Theobroma grandiflorum) Pulp and Flour
Source: Foods. 2023 May 31;12(11):2217. doi: 10.3390/foods12112217 (PMC10253220; doi:10.3390/foods12112217)
Supplement: Supplementary file 1 [file foods-12-02217-s001.zip › foods-2397047-supplementary.pdf]

Supplementary tables

Table S1. Optimism-corrected performance estimates through validation by bootstrap approach of significant models for prediction of physicochemical parameters from physicochemical variables in fermented milk beverage with cupuassu (*Theobroma grandiflorum*) pulp and flour and stored at 4 °C.

Parameter	Variable	Equation	R	R^2_{adj}	*p*	R^2_{app}	R^2_{boot}	R^2_{orig}	Optimism	R^2_v	R_v
**Storage period**	n	-0.002 *n* + 0.41	-0.32	0.105	0.012	0.548	-0.003	0.551	0.742	0.089	0.298
**Pulp**	pH	-0.03 pH + 4.47	-0.740	0.548	<0.0001	0.363	0.005	0.358	0.599	0.551	**0.742**
	Synereses	-0.24 Syn + 34.53	-0.480	0.230	0.0001	0.230	0.234	0.230	0.004	0.226	0.475
	WHC	0.24 WHC + 65.46	0.480	0.231	0.0001	0.231	0.235	0.231	0.004	0.227	0.476
	*a**	0.08 *a** + 1.51	0.430	0.189	0.001	0.189	0.183	0.188	-0.005	0.194	0.440
	K	46.91 *K* + 237.9	0.630	0.391	<0.0001	0.391	0.411	0.391	0.020	0.370	0.609
	n	-0.006 *n* + 0.42	-0.410	0.167	0.001	0.167	0.179	0.167	0.013	0.154	0.393
	App.viscosity	16.73 μ + 68.31	0.530	0.278	<0.0001	0.278	0.301	0.278	0.023	0.255	0.505
**Flour**	pH	0.08 pH + 4.180	0.6	0.364	<0.0001	0.364	0.368	0.364	0.005	0.359	0.599
	Synereses	-1.05 Syn + 34.75	-0.69	0.480	<0.0001	0.480	0.486	0.480	0.006	0.474	**0.689**
	WHC	1.05 WHC + 65.25	0.69	0.478	<0.0001	0.478	0.485	0.478	0.006	0.472	**0.687**
	*L**	-7.011 *L** + 32.83	-0.86	0.746	<0.0001	0.746	0.740	0.746	-0.006	0.752	**0.867**
	*a**	-0.19 *a** + 2.238	-0.36	0.133	0.004	0.133	0.159	0.133	0.027	0.106	0.326
	*b**	-9.52 *b** + 17.20	-0.32	0.101	0.013	0.101	0.291	0.101	0.190	-0.088	NA
	*c**	-7.82 *c** + 22.46	-0.87	0.751	<0.0001	0.751	0.745	0.750	-0.005	0.756	**0.869**
	h°	74.01 *h*° + 122.1	0.86	0.747	<0.0001	0.747	0.742	0.747	-0.005	0.753	**0.868**
**pH**	*L**	-33.49 *L** + 166.2	-0.53	0.28	<0.0001	0.280	0.290	0.280	0.011	0.269	0.519
	*a**	-2.216 *a** + 11.47	-0.53	0.283	<0.0001	0.283	0.286	0.283	0.003	0.280	0.529
	*c**	-36.26 *c** + 166.5	-0.52	0.266	<0.0001	0.266	0.275	0.265	0.010	0.256	0.505
	h°	353.1 *h*° - 1284	0.53	0.280	<0.0001	0.280	0.289	0.280	0.009	0.271	0.520
	K	-696.4 K + 3493	-0.39	0.155	0.002	0.155	0.172	0.155	0.017	0.139	0.373
	n	0.096 n - 0.02654	0.27	0.075	0.034	0.075	0.094	0.075	0.019	0.057	0.238
	App.viscosity	-270.8 μ + 1326	-0.36	0.132	0.004	0.132	0.146	0.131	0.015	0.117	0.342
**Synereses**	WHC	-0.9994 WHC + 99.98	-1	1	<0.001	0.997	0.997	0.997	0.000	0.997	**0.998**

	*L**	2.714 *L** - 67.72	0.51	0.26	<0.001	0.256	0.258	0.256	0.002	0.254	0.504
	*c**	3.015 *c** - 89.31	0.51	0.26	<0.001	0.256	0.258	0.256	0.002	0.253	0.503
	h°	-27.68 *h*° + 1152	-0.49	0.24	<0.001	0.240	0.243	0.239	0.003	0.236	0.486
**WHC**	*L**	-2.70 *L** + 203.0	-0.5	0.255	<0,0001	0.255	0.256	0.255	0.002	0.253	0.503
	*c**	-3.008 *c** + 211.7	-0.5	0.255	<0,0001	0.255	0.257	0.255	0.003	0.252	0.502
	h°	27.63 *h*° - 1613	0.49	0.239	<0,0001	0.239	0.242	0.239	0.004	0.235	0.485
L*	*a**	0.02459 *a** + 1.397	0.37	0.140	0.003	0.140	0.178	0.140	0.038	0.102	0.319
	*b**	1.670 *b** - 34.34	0.45	0.205	0.0003	0.205	0.437	0.205	0.232	-0.027	NA
	*c**	1.109 *c** - 14.02	1	0.994	<0.0001	0.994	0.994	0.994	0.000	0.994	**0.997**
	h°	-10.51 *h*° + 467.8	-1	0.994	<0.0001	0.994	0.994	0.994	0.000	0.993	**0.997**
a*	*c**	6.64 *c** - 2.19	0.39	0.154	0.002	0.154	0.192	0.153	0.039	0.115	0.340
	h°	-65.83 *h*° + 361.2	-0.41	0.169	0.001	0.169	0.204	0.167	0.036	0.132	0.364
b*	*c**	0.135 *c** + 10.33	0.45	0.201	<0.001	0.201	0.436	-0.249	0.685	-0.484	NA
	h°	-1.270 *h*° + 236.8	-0.44	0.197	<0.001	0.197	0.432	-0.255	0.687	-0.490	NA
c*	*h*°	-9.475 *h*° + 334.8	-1	0.998	<0.001	0.998	0.998	0.998	0.000	0.998	0.999
**K**	Syneresis	-0.0018 Syn + 34.09	-0.27	0.075	0.03	0.075	0.086	0.074	0.012	0.063	0.250
	WHC	0.0019 WHC + 65.89	0.28	0.076	0.03	0.076	0.088	0.076	0.012	0.064	0.253
**n**	K	-3998 K + 2047	-0.79	0.630	<0.001	0.630	0.641	0.630	0.012	0.618	**0.786**
**Apparent viscosity**	K	2.283 K + 131.0	0.97	0.932	<0.001	0.932	0.933	0.931	0.002	0.930	**0.964**
	n	-0.0003748 n + 0.447	-0.8	0.638	<0.001	0.638	0.651	-0.373	1.024	-0.387	NA

WHC: Water holding capacity; *L**: lightness; *a**: redness; *b**: yellowness; *c**: chroma; *h*°: hue angle; K: consistency index; n: flow behavior index; App.viscosity: Apparent viscosity(mPa s)**;** R: Pearson´s correlation coefficient; R^2_{adj}: adjusted coefficient of determination; *p*: probability value; R^2app: apparent coefficient of determination; R^2boot: bootstrap coefficient of determination; R^2orig: original coefficient of determination; R^2v: coefficient of determination of the model after validation. Rv: correlation coefficient of the model after validation.

Table S2. Optimism-corrected performance estimates through validation by bootstrap approach of significant models for prediction of sensory parameters from physicochemical variables in fermented milk beverage with cupuassu (*Theobroma grandiflorum*) pulp and flour and stored at 4 °C.

Sensory parameter	Variable	Equation	R	R^2_{adj}	*p*	R^2_{app}	R^2_{boot}	R^2_{orig}	Optimism	R^2_v	R_v
**Appearance**	Flour	0.67 Flour + 4.53	0.85	0.725	0.032	0.720	0.762	0.7224	0.039	0.681	0.825
	Syneresis	-0.29 Syn + 15.31	-0.87	0.757	0.024	0.760	0.821	0.748	0.073	0.687	0.829
	WHC	0.29 WHC - 13.58	0.87	0.755	0.025	0.760	0.820	0.745	0.076	0.684	0.827
**Color**	Flour	0.81 Flour + 4.26	0.94	0.89	0.005	0.890	0.899	0.8856	0.013	0.877	0.936
	Syneresis	-0.32 Syn + 16.31	-0.88	0.777	0.020	0.780	0.843	0.763	0.080	0.700	0.837
	WHC	0.32 WHC - 15.55	0.88	0.774	0.021	0.770	0.842	0.760	0.082	0.688	0.829
**Flavor**	Flour	0.34 Flour + 5.49	0.83	0.695	0.039	0.690	0.740	0.6943	0.046	0.644	0.803
	Syneresis	-0.15 Syn + 10.91	-0.86	0.743	0.027	0.740	0.801	0.739	0.063	0.677	0.823
	WHC	0.1451WHC - 3.625	0.86	0.740	0.028	0.740	0.800	0.735	0.064	0.676	0.822
**Alcoholic(JAR)**	Pulp	0.02780 Pulp + 2.942	0.85	0.714	0.034	0.710	0.717	0.7083	0.009	0.701	0.837
	*b**	-0.3603 *b** + 2.297	-0.88	0.781	0.020	0.780	0.789	0.775	0.014	0.766	0.875
**Cupuassu JAR**	pH	-1.488 pH + 9.356	-0.93	0.871	0.007	0.870	0.733	0.833	-0.100	0.970	0.985
	*L**	0.2730 *L** - 1.093	0.87	0.756	0.025	0.760	0.728	0.742	-0.014	0.774	0.880
**Sweet JAR**	Flour	-0.2736 Flour + 2.591	-0.89	0.788	0.018	0.790	0.793	0.7851	0.008	0.782	0.884
**Bitter(JAR)**	Flour	0.2769 Flour + 2.929	0.94	0.875	0.006	0.870	0.879	0.8702	0.009	0.861	0.928
	Syneresis	-0.1020 Syn + 6.811	-0.82	0.670	0.047	0.670	0.753	0.658	0.095	0.575	0.758
	WHC	0.1014 WHC - 3.348	0.82	0.668	0.047	0.670	0.753	0.654	0.098	0.572	0.756
**White(JAR)**	Flour	-0.2438 Flour + 3.398	-0.97	0.934	0.002	0.930	0.932	0.9319	0.000	0.930	0.964
	Syneresis	0.1014 Syn - 0.3917	0.96	0.912	0.003	0.910	0.935	0.907	0.028	0.882	0.939
	WHC	-0.1009 WHC + 9.714	-0.95	0.910	0.003	0.910	0.935	0.905	0.030	0.880	0.938
**Brown(JAR)**	Flour	0.2878 Flour + 2.202	0.96	0.930	0.002	0.930	0.935	0.9276	0.007	0.923	0.961
	Syneresis	-0.1108 Syn + 6.391	-0.88	0.777	0.020	0.780	0.822	0.769	0.053	0.727	0.853
	WHC	0.1101 WHC - 4.643	0.88	0.775	0.021	0.770	0.821	0.766	0.055	0.715	0.846
**Cupuassu(JAR)**	Pulp	0.06890 Pulp + 2.644	0.84	0.708	0.036	0.710	0.735	0.7044	0.031	0.679	0.824
	*c**	1.785 *c** - 2.060	0.9	0.805	0.015	0.810	0.793	0.804	-0.011	0.821	0.906
**Mouthfeel (JAR)**	*a**	-0.7049 *a** + 3.856	-0.83	0.691	0.040	0.690	0.702	0.674	0.028	0.662	0.813
	K	0.0001972 K + 2.500	0.94	0.879	0.006	0.880	0.800	-1.180	1.980	-1.100	NA
	App.viscosity	0.0004728 μ + 2.518	0.9	0.810	0.015	0.810	0.707	0.627	0.080	0.730	0.854

WHC: Water holding capacity; *L**: lightness; *a*:* redness; *b*:* yellowness; *c**: chroma; *h*°: hue angle; K: consistency index; n: flow behavior index; App.viscosity: Apparent viscosity(mPa s)**;** R: Pearson´s correlation coefficient; R^2_{adj}: adjusted coefficient of determination; *p*: probability value; R^2app: apparent coefficient of determination; R^2boot: bootstrap coefficient of determination; R^2orig: original coefficient of determination; R^2v: coefficient of determination of the model after validation. Rv: correlation coefficient of the model after validat

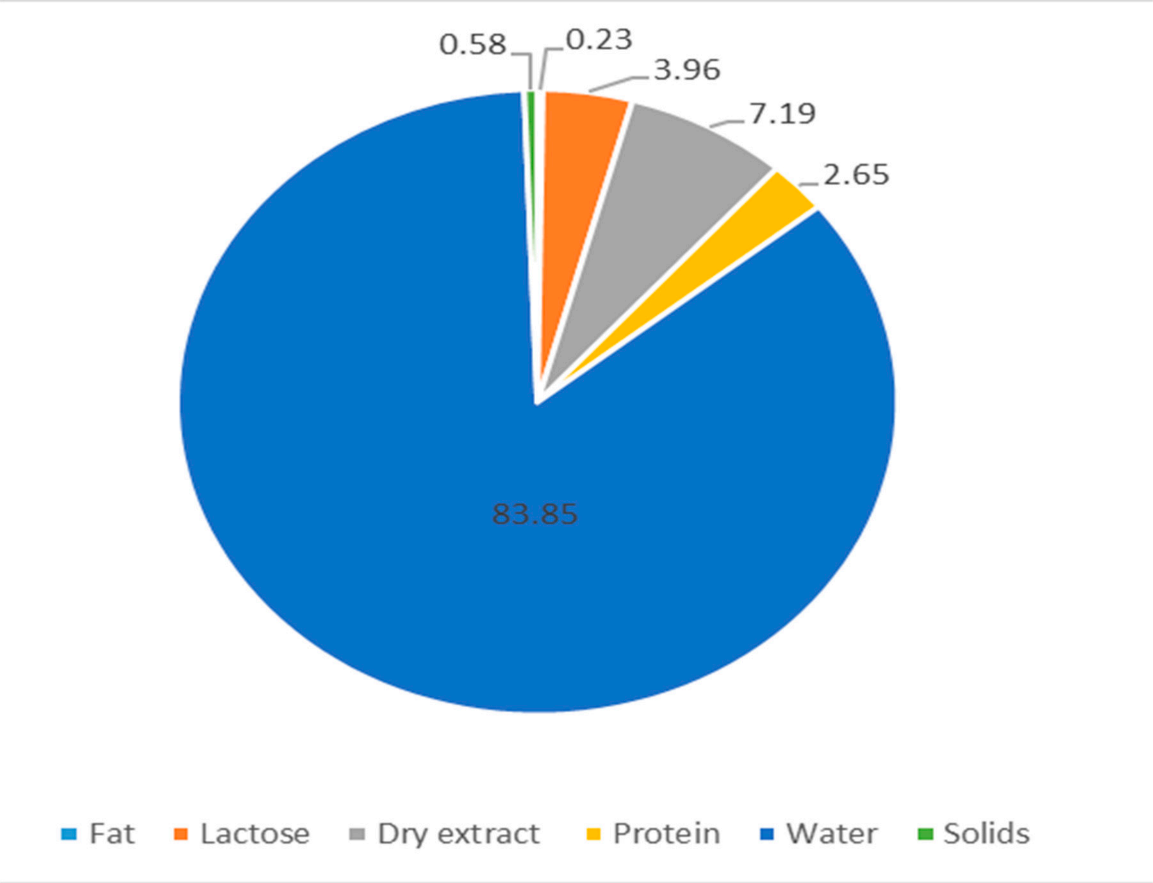

Figure S1. Proximal composition of whey used to prepare artisanal fermented milk beverages.

Supplementary figures

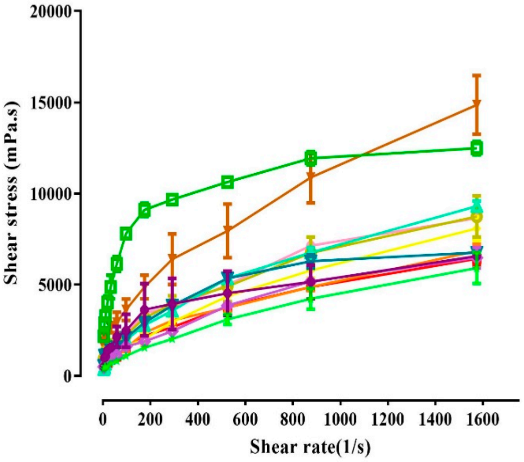

(a)

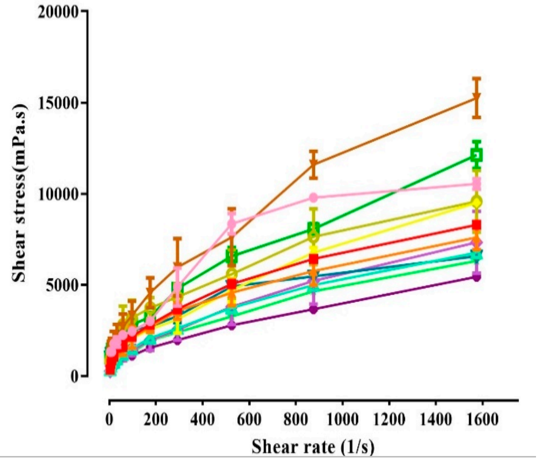

(b)

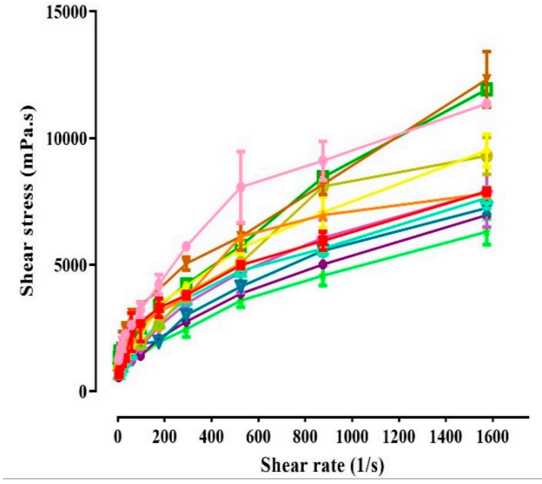

(c)

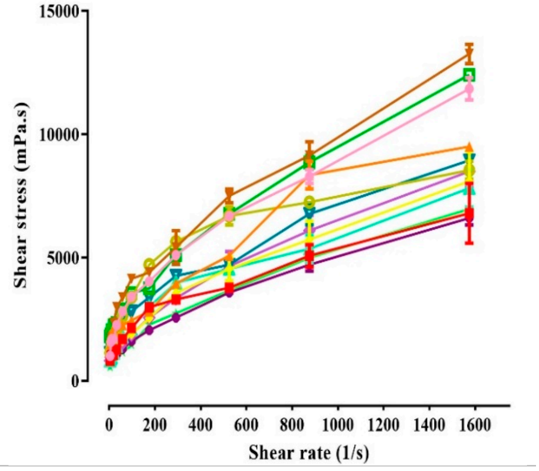

(d)

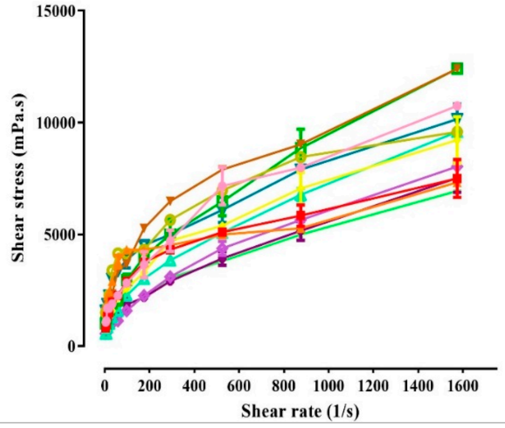

(e)

Figure S2. Effect of shear rate on shear stress of fermented milk beverage : ( ) 10% pulp, ( ) 5% pulp, ( ) 7.5% pulp, ( ) 10% pulp and 3% flour, ( ) 5% pulp and 3% flour, ( ) 7.5% pulp and 3% flour, ( ) 10% pulp and 1.5% flour, ( ) 5% pulp and 3% flour, ( ) 7.5% pulp and 3% flour, ( ) control, ( ) 3% flour, ( ) 1.5% flour during storage a) day 0; b) day 7; c) day 14; d) day 21 and e) day 28.

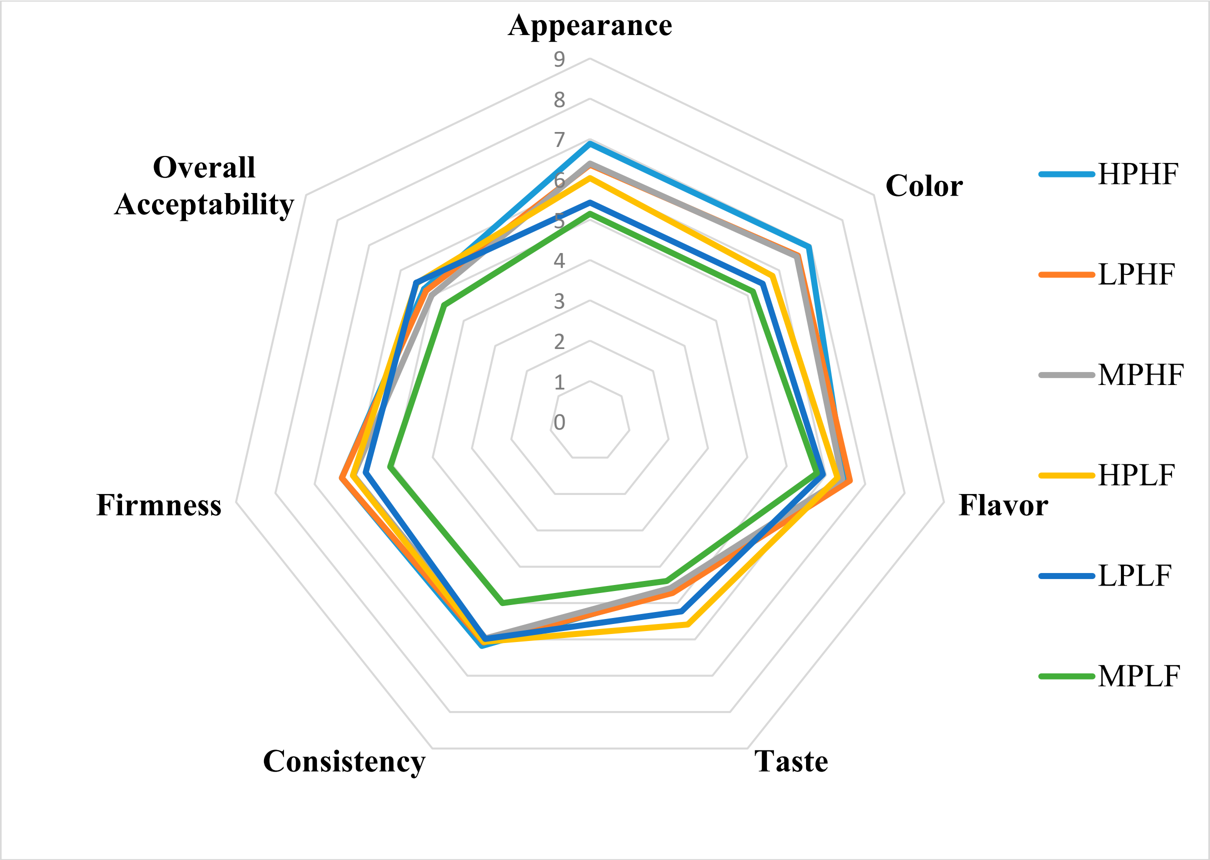

Figure S3. Spider plot of mean scores of sensory evaluation of fermented milk beverage with cupuassu flour and pulp. HPHF, beverage (10% pulp and 3% flour); LPHF, beverage (5% pulp and 3% flour); MPHF, beverage (7.5% pulp and 3% flour); HPLF, beverage (10% pulp and 1.5% flour); LPLF, beverage (5% pulp and 1.5% flour); MPLF, beverage (7.5% pulp and 1.5% flour).

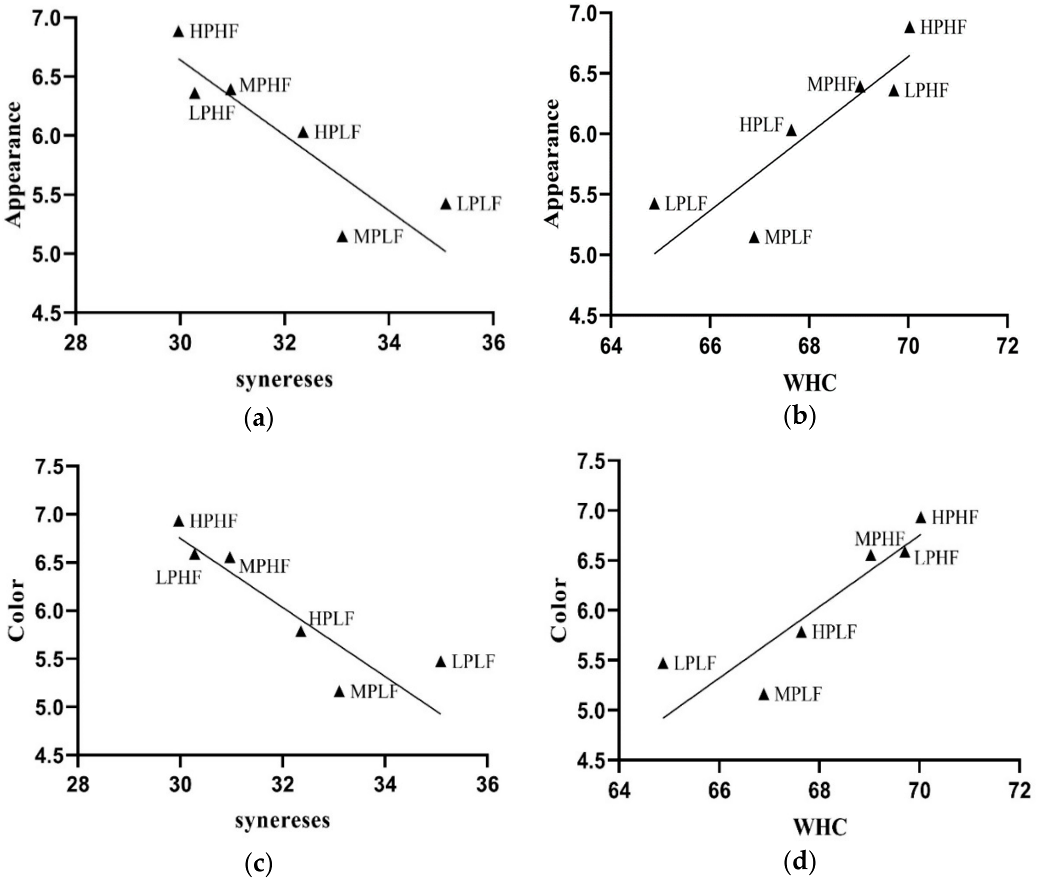

Figure S4. Significant correlations ($p < 0.05$) and internally validated by Bootstrap method of sensory attributes in relation to physicochemical parameters of fermented milk beverages stored at 4 °C. a) Appearance: syneresis; b) Appearance: water holding capacity (WHC); c) color: syneresis; d) color: water holding capacity (WHC).

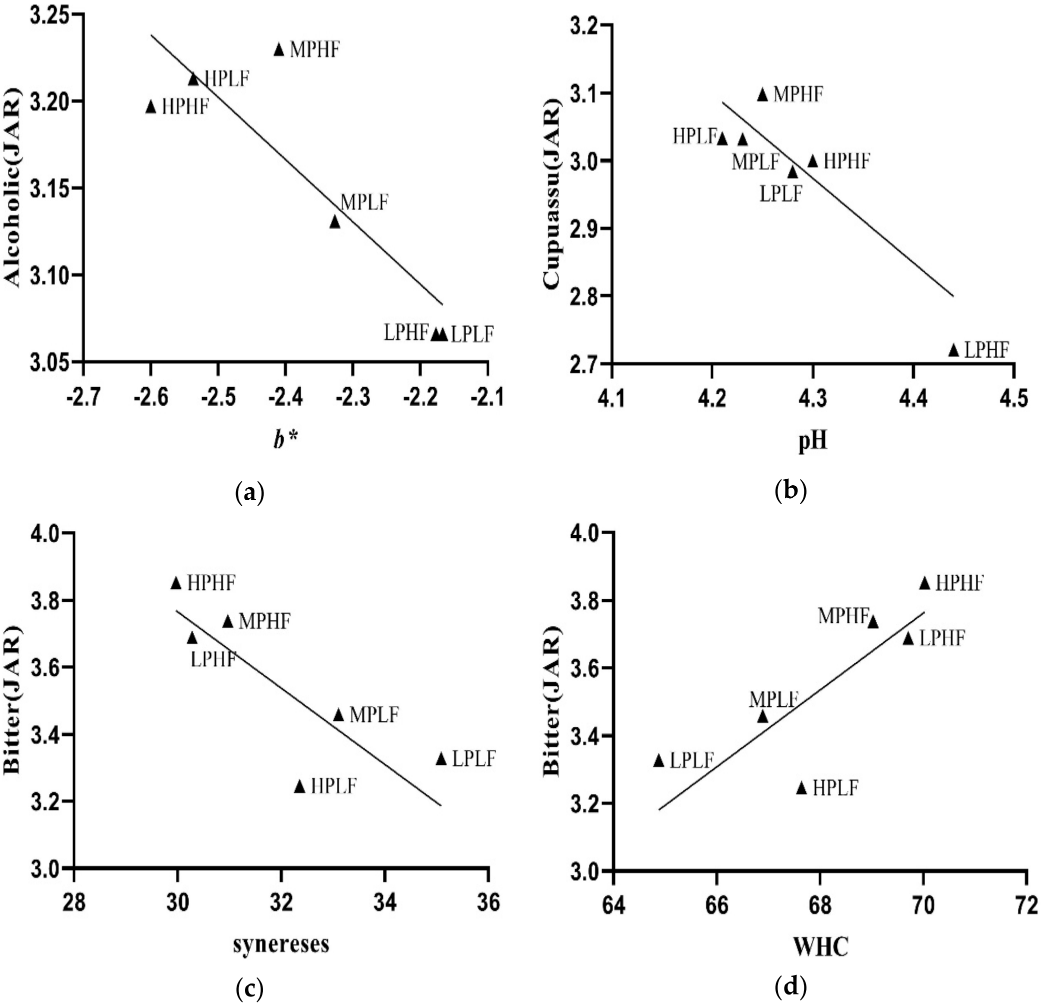

Figure S5. Significant correlations ($p < 0.05$) and internally validated by Bootstrap method of sensory attributes in relation to physicochemical parameters of fermented milk beverages stored at 4 °C. a) Alcoholic (JAR): *b**; b) cupuassu (JAR): pH; c) Bitter (JAR): syneresis; d) Bitter (JAR): water holding capacity (WHC).

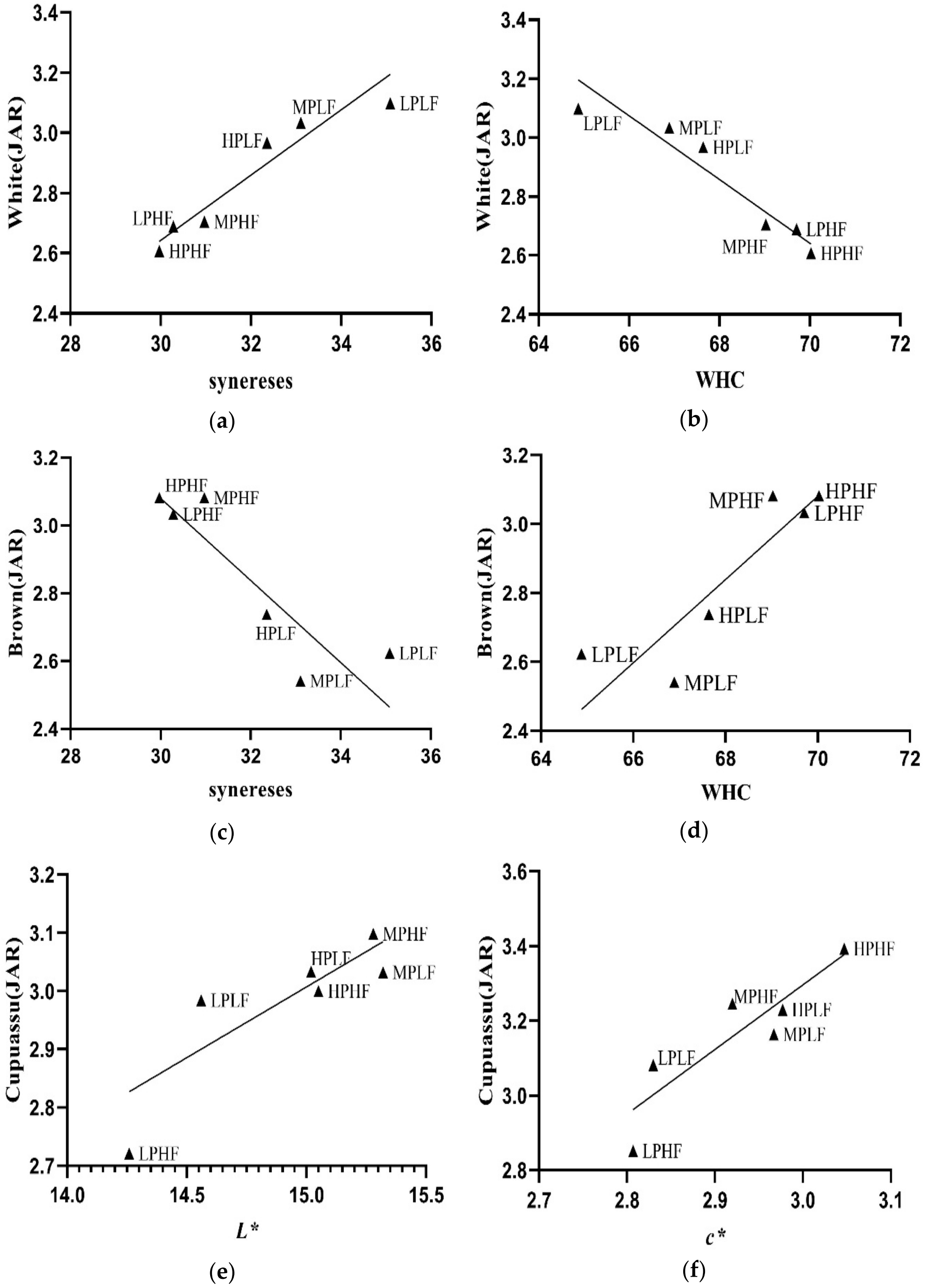

Figure S6. Significant correlations ($p < 0.05$) and internally validated by Bootstrap method of sensory attributes in relation to physicochemical parameters of fermented milk beverages stored at 4 °C. a) White (JAR): syneresis; b) White (JAR): water holding capacity (WHC); c) Brown (JAR): syneresis d) Brown (JAR): water holding capacity (WHC); e) Cupuassu (JAR): *L**; f) Cupuassu (JAR): *c**.

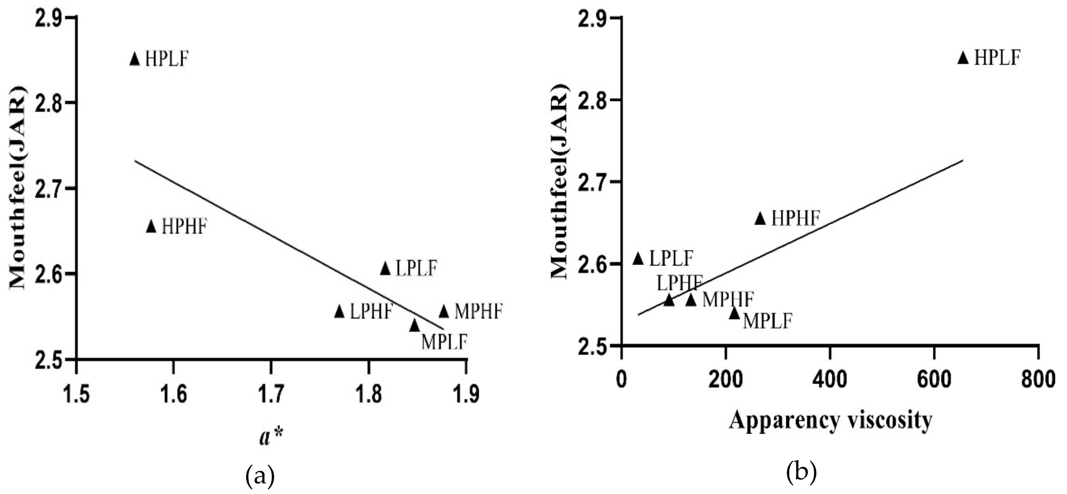

Figure S7. Significant correlations ($p < 0.05$) and internally validated by Bootstrap method of sensory attributes in relation to physicochemical parameters of fermented milk beverages stored at 4 °C. a) Mouthfeel (JAR): *a**; b) Mouthfeel (JAR): Apparency viscosity.
